# Gut Microflora Modulates Th17/Treg Cell Differentiation in Experimental Autoimmune Prostatitis *via* the Short-Chain Fatty Acid Propionate

**DOI:** 10.3389/fimmu.2022.915218

**Published:** 2022-07-04

**Authors:** He-Xi Du, Shao-Yu Yue, Di Niu, Chang Liu, Li-Gang Zhang, Jing Chen, Yang Chen, Yu Guan, Xiao-Liang Hua, Chun Li, Xian-Guo Chen, Li Zhang, Chao-Zhao Liang

**Affiliations:** ^1^Department of Urology, The First Affiliated Hospital of Anhui Medical University, Anhui Medical University, Hefei, China; ^2^Institute of Urology, Anhui Medical University, Hefei, China; ^3^Anhui Province Key Laboratory of Genitourinary Diseases, Anhui Medical University, Hefei, China; ^4^Department of General Surgery, The First Affiliated Hospital of Anhui Medical University, Hefei, China

**Keywords:** prostatitis, Th17, Treg, gut dysbiosis, propionic acid

## Abstract

Chronic prostatitis/chronic pelvic pain syndrome (CP/CPPS) is a very common urological disorder and has been gradually regarded as an immune-mediated disease. Multiple studies have indicated that the gut microflora plays a pivotal part in immune homeostasis and autoimmune disorder development. However, whether the gut microflora affects the CP/CPPS, and the underlying mechanism behind them remain unclear. Here, we built an experimental autoimmune prostatitis (EAP) mouse model by subcutaneous immunity and identified that its Th17/Treg frequency was imbalanced. Using fecal 16s rRNA sequencing and untargeted/targeted metabolomics, we discovered that the diversity and relative abundance of gut microflora and their metabolites were obviously different between the control and the EAP group. Propionic acid, a kind of short-chain fatty acid (SCFA), was decreased in EAP mice compared to that in controls, and supplementation with propionic acid reduced susceptibility to EAP and corrected the imbalance of Th17/Treg cell differentiation *in vivo* and *in vitro*. Furthermore, SCFA receptor G-protein-coupled receptor 43 and intracellular histone deacetylase 6 regulated by propionic acid in Th17 and Treg cells were also evaluated. Lastly, we observed that fecal transplantation from EAP mice induced the decrease of Treg cell frequency in recipient mice. Our data showed that gut dysbiosis contributed to a Th17/Treg differentiation imbalance in EAP *via* the decrease of metabolite propionic acid and provided valuable immunological groundwork for further intervention in immunologic derangement of CP/CPPS by targeting propionic acid.

## Introduction

Prostatitis is a common urogenital disease related with high morbidity and recurrence rate, accounting for approximately one-quarter of all visits in urology clinics (1). Chronic prostatitis/chronic pelvic pain syndrome (CP/CPPS), also named Type III chronic prostatitis, is the most common type of prostatitis, which accounts for about 90%–95% of all population of prostatitis (2). Featured with the long-term chronic pain in the pelvic area with or without lower urinary symptoms, CP/CPPS severely influences the patients’ quality of life and has become a global recognized threat to male health. The etiology of CP/CPPS is still unidentified, which leads to a limited availability of its treatment methods. Although several factors, such as urinary reflux, neurological disorder, and endocrine abnormality, have been previously reported to relate to CP/CPPS, accumulating lines of evidence in recent years have demonstrated a vital role for immunological imbalance in its pathogenesis (3). Thus, CP/CPPS has also been considered as an organ-specific autoimmune disease (4, 5). Nevertheless, it is imperative to sharpen our understanding on the exact immunological mechanisms of CP/CPPS, and to explore pertinent treatments.

T lymphocytes, particularly CD4^+^ T cells, are found to be major contributors to the pathogenesis of CP/CPPS as well as its most commonly used animal model, experimental autoimmune prostatitis (EAP) (6, 7). The Th17 cells are one subset of CD4^+^ T cells, characterized by the release of cytokine interleukin-17 (IL-17). It was reported that Th17 cells were closely associated with the occurrence of multiple autoimmune disorders (8). As for CP/CPPS, Motrich et al. (9) found that Th17 cells’ immune response was related to chronic inflammation in the male genital tract, which might be the foundation for the induction and aggravation of pelvic pain and male reproductive tract inflammation. In animal experiments, it was also displayed that IL-17 could mediate pelvic pain in EAP mice (10). Regulatory T cells (Treg cells), another subgroup of CD4^+^ T cells, belong to immunosuppressive cells that are conducive to maintaining immune tolerance. They suppress various immune pathologies through the production of inhibitory cytokines, such as transforming growth factor-β (TGF-β) and interleukin-10 (IL-10) (11). The elimination of Treg cells can increase the morbidity and severity of autoimmune disorders. We have previously described that the percentages of Treg cells in the peripheral blood of patients with CP/CPPS and in prostate of EAP mice were markedly reduced (12, 13). Nevertheless, the regulatory mechanisms of the Th17 and Treg cell differentiation in EAP are still poorly understood.

Gut microflora refers to various parasitic microorganisms in the intestinal tract of organisms. It exerts several immunomodulatory functions and has been recognized to play an indispensable role in modulating the immune/inflammatory responses of host over the past two decades. Studies have linked gut microflora dysbiosis with the increased prevalence of multiple autoimmune diseases, such as systematic lupus erythematosus (SLE) and autoimmune thyroiditis (14, 15). The changes in microbiome have also been reported to relate to CP/CPPS, and their relationship has been emphasized gradually by the academic community (16). Our previous study has further identified that gut microflora maladjustment is related to EAP-induced depressive behaviors in mice (17). Generally, systemic functions of the gut microflora are found to be largely attributed to microbial metabolome, especially short-chain fatty acids (SCFAs), the most well-characterized final products of dietary fiber fermentation (18). On one hand, these bacterially derived SCFAs can enter the circulation to regulate the immune system, including Th17 and Treg cells, in extra-intestinal organs (19). On the other hand, SCFAs have been demonstrated to promote immune cells in the intestine to enter the circulation and remote tissues (20). Thus, the relationships between SCFA metabolisms and immunological disorders have been confirmed and accepted. It is not known whether gut microbiota-driven SCFAs exert effects on prostatitis and, if so, what the mechanism is.

In this study, we deeply explored the impact of gut microflora on the regulation of immune responses in an EAP mouse model *via* its metabolites. We first built EAP models and detected the percentages of Th17 and Treg cells and the concentrations of their associated inflammatory factors. Then we analyzed gut microbiome profiles and the metabolic products in EAP mice in order to investigate whether abundance of gut microbiota and associated metabolites was altered in EAP. Of special interest, we have found that propionic acid, a kind of SCFA, was significantly decreased in the EAP group. Furthermore, we investigated whether propionic acid supplementation might be an effective treatment in prostatic inflammation and Th17/Treg cell differentiation imbalance, and explored the biological mechanisms underlying them through *in vitro* and *in vivo* experiments. Our study emphasizes the functional influences of the gut microflora-driven metabolites on Th17/Treg cell differentiation in an EAP model.

## Materials and Methods

### Animals

Male non-obese diabetic (NOD) mice (age = 5 weeks; weight = 17 ± 2 g) were subscribed from the Nanjing Model Animal Center (Nanjing, China) and fed in the specific pathogen-free (SPF) laboratory animal room of Anhui Medical University. All animal experiments were designed and conducted following the guidelines of the Institutional Animal Care and Use Committee of Anhui Medical University.

### Mouse Model of EAP

The EAP mouse model was built according to our previously reported method (21). In short, mice in the EAP group were immunized by subcutaneous injection into three sites, including the tail root (0.050 ml), bilateral foot pads (0.025 ml for one side), and mouse shoulder (0.050 ml), with emulsion consisting of equal volumes of rat male accessory gland extracts and complete Freund’s adjuvant (CFA, Sigma). Two weeks after the first immunization, mice were immunized again by injection of male accessory gland extracts emulsified with equal volumes of incomplete Freund’s adjuvant (IFA, Sigma). In the corresponding period, control mice were dealt with 0.9% saline rather than prostate antigen. Mice in each group were tested on their pelvic pain by using an apparatus of Von Frey filaments (North Coast Medical, USA).

### Histopathology and Immunohistochemistry

To identify the presence of prostatic inflammation, prostate glands of mice were adequately fixed in 10% paraformaldehyde solution, embedded by paraffin, and cross-cut into 4-μm slices. Then, the prostate sections were dyed with hematoxylin–eosin (HE) and successively scored for the inflammatory level by using a previously reported grading principle (0–3 stand for the degree of the prostatitic inflammation, 0 for non-inflammation and 3 for apparent inflammatory cell infiltration) (22).

Immunohistochemistry was also performed on paraffin-embedded prostate sections. The slices were separately de-paraffinized in xylene and incubated in a graded series of ethanol (100%, 85%, and 75%). After antigen retrieval by EDTA antigen repair buffer and quenching of endogenous peroxidase, the sections were stained with primary rabbit anti-G-protein-coupled receptor 43 (GPR43, Genetex, 00823, USA) antibody at 1:200, rabbit anti-IL-17 (Servicebio, China) antibody at 1:200, rabbit anti-forkhead box P3 (FoxP3, Servicebio, China) antibody at 1:150, mouse anti-histone deacetylase 6 (HDAC6, Genetex, 84377, USA) at 1:150, and appropriate secondary antibodies (goat anti-rabbit/mouse IgG; Servicebio, China) at 1:200. The images of sections were captured with a digital slice scanner (Pannoramic MIDI, 3DHistech, Hungary).

### Flow Cytometry

The proportions of Th17 and Treg cells were detected using flow cytometry. In brief, lymphocytes were isolated from the spleens of mice and washed twice by phosphate-buffered saline (PBS) solution. Subsequently, the cells were transferred to test tubes and incubated with surface antibody of FITC-labeled CD4 (rat anti-mouse, BD, 553046, USA) and APC-labeled CD25 (rat anti-mouse, BD, 557192, USA). After being washed again by PBS, 1 ml of 1640 medium (Gibco, USA) containing phorbol 12-myristate 13-acetate (PMA, MultiSciences, China), lonomycin (MultiSciences, China), and monensin (MultiSciences, China) was added in each tube, incubated at 37°C for 4 h, and washed again. Next, cells were fixed and permeated successively, followed by incubating with intracellular antibody, including PE-labeled IL-17A (rat anti-mouse, BD, 559502, USA), PE-labeled retinoic acid−related orphan receptor γt (RORγt, rat anti-mouse, ebioscience, 12-6981-60, USA), and PE-labeled FoxP3 (rat anti-mouse, BD, 563101, USA), at a temperature of 4°C for 60 min. After being washed again and analyzed by a FACSCalibur flow cytometer (Beckman Coulter, USA), the resulting data were processed by FlowJo software (Tree Star, Ashland).

### Isolation and *In Vitro* Differentiation of Naive CD4^+^ T Cells

Isolation of naive CD4^+^ T cells from the mice spleen and differentiation into Th17/Treg cells *in vitro* were conducted according to our previously reported method with minor modifications (23). CD4^+^ T cells from splenic lymphocyte were magnetically enriched by a CD4^+^ T Cell Isolation Kit (Miltenyi, 130-104-454, Germany) with Columns (LS Column, Miltenyi, 130-042-401). Sorted naive CD4^+^ T cells were cultured for 5 days in 24-well plates in 1640 medium (including 10% fetal bovine serum and 1% penicillin–streptomycin solution) before supplemented with 10 μg/ml anti-CD3 (Bio X Cell, BE0001-1, USA) and 10 μg/ml anti-CD28 (Bio X Cell, BE0015-1). Th17 stimulations were complemented with 40 ng/ml IL-6 (Novoprotein, CG39), 1 ng/ml TGF-β1 (Novoprotein, CA59), 40 ng/ml IL-23 (Novoprotein, CS31), 20 μg/ml anti-IL-4 (Bio X Cell, BE0045), and 20 μg/ml anti-IFN-γ (Bio X Cell, BE0055). Treg stimulations were supplemented with 5 ng/ml TGF-β1 (Novoprotein, CA59), 20 ng/ml IL-2 (Novoprotein, CK24), 10 μg/ml anti-IL-4 (Bio X Cell, BE0045), and 10 μg/ml anti-IFNγ (Bio X Cell, BE0055).

### Lentivirus Infection

Gene suppression of GPR43 was conducted with the short hairpin RNA (shRNA) by using LV as vector. LV packaging with the GPR43 shRNA (LV-Sh GPR43) and empty vector (LV-Sh Control) was subscribed from Hanbio Biotechnology Co., Ltd. (Shanghai, China, HH20211029RFF-LV01). The target sequence was 5′-CCCATGGCAGTCACCATCTTCTGTT-3′. To generate stable gene knockdown cells, LV-shRNAs were transfected in strict accordance with the manufacturer’s instructions. Suppression was confirmed using the Western blot method.

In mice, intravenous injection of LV-Sh GPR43 or LV-Sh Control was implemented 2 days before EAP induction. On the 14th day after EAP induction, pentobarbital sodium (60–80 mg/kg) was administered by intraperitoneal injection for the anesthesia of mice. The lower abdomens of mice were incised under sterile conditions, and the prostate was exposed. The LV solution was injected slowly into the bilateral coagulating glands and ventral and dorsolateral lobes of prostate (75 μl/mouse). After that, the wound was closed.

### Enzyme-Linked Immunosorbent Assay

The concentrations of cytokines including IL-17, IL-10, TGF-β, granulocyte-macrophage colony stimulating factor (GM-CSF), and interferon gamma (IFNγ) in serum and the levels of IL-17, IL-10, and TGF-β in the supernatant of the prostate tissues were measured by the ELISA method. In terms of detection of cytokines from the prostate tissues, samples were washed, weighed at a dose of 10 mg, and cut into pieces, and 90 μl of PBS was added. The mixtures were homogenized in a glass homogenizer. After centrifugation, the supernatants were obtained for detection. All experiments were performed by using the corresponding ELISA Kit (Elabscience, China) based on the manufacturer’s protocol.

### DNA Extraction and 16S rRNA Sequencing

Feces were collected and frozen immediately. The bacterial genomic DNA of fecal samples was extracted with the QIAamp 96 PowerFecal QIAcube HT kit (QIAGEN, Germany). The V3–V4 region amplicon sequencing 343F (5’ TACGGRAGGCAGCAG 3’) and 798R (5’ AGGGTATCTAATCCT 3’) of the 16S rRNA gene were performed by an Illumina Miseq PE300 system (Illumina Company, USA). Raw sequencing data were in FASTQ format, and then bioinformatic analysis was carried out. Trimmomatic software was applied to detect the paired-end reads and cut off ambiguous bases (N). Low-quality sequences (quality score is less than 20 on average) were cut out by using a sliding window trimming approach, followed by an assembly by FLASH software. Then, QIIME software (version 1.8.0) was applied to further de-noise the sequences. Next, using usearch software, primer sequences were removed and clustered with 97% similarity threshold to generate operational taxonomic units (OTU). The representative read for each OTU was assigned by the QIIME package, and finally annotated and blasted against the Silva database (version 123) by the RDP classifier with a 70% confidence threshold. The analyses for all parameters such as α-diversity and β-diversity of microbes were performed on the basis of using the OECloud platform (https://cloud.oebiotech.cn/task/detail/micro-pipline-oehw/) developed by OE Biotech Co., Ltd. (Shanghai, China).

### Metabolome Sequencing of Feces

The metabolites of feces were isolated and extracted as follows. First, 60-mg fecal samples were precipitated with 360 µl of methanol. All samples were processed to grind and ultrasound extraction. After 200 μl of chloroform was added, the mixtures were vortexed. Samples were then centrifuged at 12,000 rpm for 10 min, and the acquired supernatant was transferred and vacuum-dried. Subsequently, an 80-μl solution of methoxyamine hydrochloride pyridine, 80 μl of BSTFA derivatization reagent, and 20 μl of hexane were successively added.

The pretreated samples were next subjected to metabolomics profiling by a gas chromatography detection system (Agilent 7890B, Agilent Technologies, USA) with an MSD system (Agilent 5977A, Agilent Technologies, USA). Gas chromatography-mass spectrometry (GC-MS) raw data were transferred into.abf format and analyzed by the MD-DIAL software for processing. The metabolites were annotated *via* the LUG database. In the “data array”, internal standards and pseudo positive peaks were eliminated. After deleting the internal label RSD > 0.3, all peak strengths were processed by normalization of multi-interior label in the light of retention time partition period. The resulting data matrix were subsequently imported to R ropls package for analysis. After mean centering and unit variance scaling, principal component analysis (PCA) and orthogonal partial least-squares discriminant analysis OPLS-DA were used to identify the differential metabolites among groups. T2 region of Hotelling defines the 95% confidence interval of the modeling variable. Variable importance in the projection (VIP) ranks the total contribution of each variable to the OPLS-DA model, and VIP > 1 is regarded to be related to group discrimination. The selection of differential metabolites was on the basis of the combination of the VIP values acquired from the OPLS-DA and *p*-values obtained from a Student’s *t*-test (two-tailed) on the normalized peal areas from samples in different groups. The analyses for all parameters such as volcano plot and hierarchical clustering heatmap of metabolites were performed on the basis of using the OECloud platform (https://cloud.oebiotech.cn/task/detail/meta-luming-oehw/) developed by OE Biotech Co, Ltd (Shanghai, China).

### SCFAs Measurement Assay

The GC-MS and liquid chromatograph mass spectrometer (LC-MS) were applied for the measurement of SCFA concentrations in feces and serum, respectively. For feces, 150 mg of samples was weighed, and 1 ml of 5 mmol/L NaOH solution was added. The samples were homogenized for 3 min and extracted by ultrasound for 7 min. After centrifugation, 500 μl of supernatants was transferred to a 4-ml injection glass bottle. The derivatization of samples and standards was then carried out, and GC-MS was performed to determine the SCFA concentrations. For plasma samples, 150 μl of 50% acetonitrile aqueous solutions (V/V) (including [2H9]-pentanoic acid and [2H11]-hexanoic acid) was added to 150 μl of plasma. After ultrasonic extraction for 10 min and centrifugation at 12,000 rpm for 10 min, 80 μl of supernatants was transferred into the injection vial. Next, the derivatization of samples and standards was performed, and LC-MS was utilized to determine the SCFA concentrations by high-performance liquid chromatography (HPLC, Nexera UHPLC LC-30A, Japan) and a highly sensitive mass spectrometer (AB Sciex Qtrap 5500, USA).

### SCFA Propionate Treatment Assays

After EAP induction, a group of mice were treated with propionic acid (Sigma, USA) added into drinking water (150 mM, fresh solutions four times a week) for 7 days, as described previously (24). In *in vitro* experiments, sorted naive CD4^+^ T cells were stimulated under Th17 or Treg cell conditions with or without propionic acid for 5 days at concentrations of 10 μg/ml.

### Western Blot Analysis

The cultured CD4^+^ T cells under Th17/Treg differentiation conditions were lysed in RIPA lysate (Thermo Scientific, USA) plus protease inhibitors (Beyotime, China). Proteins were subjected to electrophoresis separation and then transferred to polyvinylidene fluoride membranes. The membranes were incubated with 5% milk for 1 h, followed by incubating with the GPR43 antibody (1:800, Genetex) overnight at 4°C. GAPDH (1:5,000, Elabscience) was applied as a loading control. The membranes were washed in TBST, incubated with a corresponding secondary antibody (anti-rabbit or anti-mouse IgG, 1:5,000, Elabscience) for 1 h, and washed again by TBST. The protein bands were visualized by an enhanced chemiluminescence (ECL) system (ChemiScope 5600; Hengmei Technology, China).

### Real-Time Polymerase Chain Reaction

Total RNA was extracted from the isolated or cultured cells by TRIzol reagent (Invitrogen, USA) based on the manufacturer’s instructions. The synthesis of first-strand cDNA was conducted with a FastQuant RT Kit (Tiangen Biotech, China) and reverse transcribed by a SuperReal PreMix Plus Kit (Tiangen Biotech). All qPCRs were run on an ABI 7500 PCR system (Applied Biosystems, USA). The primers used were as follows: HDAC6 (F 5′ TCCACCGGCCAAGATTCTTC 3′; R 5′ GCCTTTCTTCTTTACCTCCGCT 3′), T-box expressed in T cells (T-bet, F 5′ AGCAAGGACGGCGAATGTT 3′; R 5′ GGGTGGACATATAAGCGGTTC 3′), and GATA binding protein 3 (GATA3, F 5′ CTCGGCCATTCGTACATGGAA 3′; R 5′ GGATACCTCTGCACCGTAGC 3′). All reactions were performed in a 20-µl tube, and mouse GAPDH was applied as an internal standard. The formula 2^−ΔΔCt^ method was applied for each sample during data processing.

### Pseudo Germ-Free Mice Modeling and Fecal Microbiota Transplantation

Fecal samples of mice were applied to colonize the intestines of antibiotic-treated mice. First, a group of male NOD mice (used as recipient mice) were treated with a cocktail of antibiotics, including 1 g/L neomycin sulfate (Sigma), 1 g/L ampicillin (Sigma), and 1 g/L metronidazole (Sigma), to build a pseudo germ-free mice model as described previously (17). Then, fecal samples from control and EAP mice were separately collected, diluted (every 1 g stool in 10 ml of sterile PBS), and administered by gavage into the antibiotic-treated pseudo-germ-free mice for 2 weeks.

### Statistical Analysis

All results were expressed as the mean ± standard deviation (SD). SPSS software version 21.0 (IBM Corp, Armonk, NY, USA) was used for statistical analysis. Two-tailed Student’s *t*-tests or Wilcoxon rank sum test was applied for the comparison of two groups. Results were considered statistically significant if *p*-values < 0.05.

## Results

### Significant Abnormalities in the Proportion of Th17 and Treg Cells and Relative Cytokine Levels of EAP

**Figure 1:**
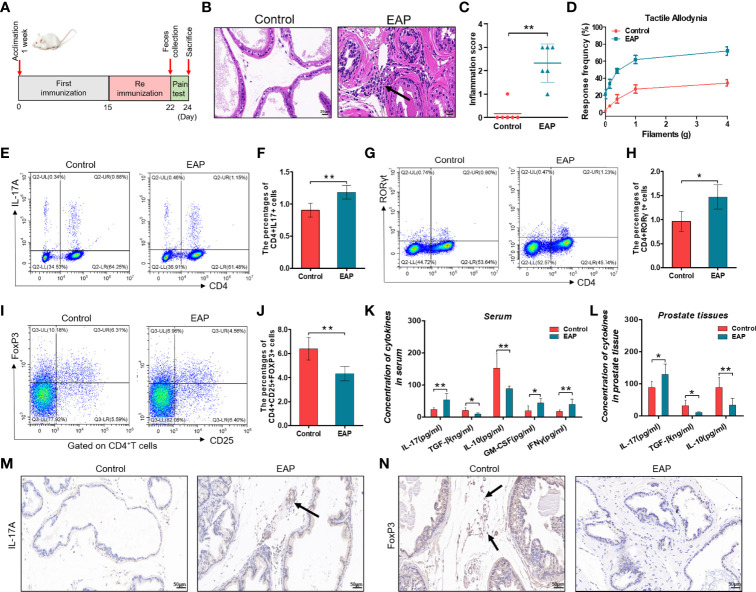
Evaluation of the EAP model, proportion of Th17/Treg cells, and levels of related inflammatory cytokines. **(A)** After 1 week of acclimation, mice received primary and secondary immunization on days 0 and 15, respectively. Modeling was completed 1 week later, followed by feces collection and pain measurement for 2 days. Mice were sacrificed on day 24 and then samples were collected. **(B)** Representative HE staining images of prostate tissue sections of the control and EAP group. The black arrowhead indicates infiltration of inflammatory cells in the EAP group. **(C)** Inflammation score of prostate in the control and EAP group. **(D)** EAP induced pelvic pain as evaluated by tactile allodynia. **(E)** Representative pictures of flow cytometric staining for CD4^+^IL-17^+^ cells in the splenic lymphocytes from control and EAP mice. **(F)** Flow cytometric analysis of the proportion of CD4^+^IL-17^+^ cells in control and EAP mice. **(G)** Representative pictures of flow cytometric staining for CD4^+^RORγt^+^ cells in the splenic lymphocytes from control and EAP mice. **(H)** Flow cytometric analysis of the proportion of CD4^+^RORγt^+^ cells in control and EAP mice. **(I)** Representative pictures of flow cytometric staining for CD4^+^CD25^+^FoxP3^+^ cells in the splenic lymphocytes from control and EAP mice. **(J)** Flow cytometric analysis of the proportion of CD4^+^CD25^+^FoxP3^+^ cells in control and EAP mice. **(K)** Serum concentrations of IL-17, TGF-β, IL-10, GM-CSF, and IFNγ by ELISA in the control and EAP group. **(L)** Concentrations of IL-17, TGF-β, and IL-10 in 10-mg prostate tissues in the control and EAP group. **(M)** Immunohistochemical staining of IL-17A (marker of Th17) on prostate sections in the control and EAP group. **(N)** Immunohistochemical staining of FoxP3 (marker of Treg) on prostate sections in the control and EAP group. The black arrows indicate the IL-17/FoxP3-positive cells in the two groups. Data are from one experiment representative of three independent experiments. *n* = 5–6/group; *p < 0.05; **p < 0.01; EAP, experimental autoimmune prostatitis; HE, hematoxylin–eosin; IL-17, interleukin-17; RORγt, retinoic acid−related orphan receptor γt; GM-CSF, granulocyte-macrophage colony stimulating factor; IFNγ, interferon gamma; TGF-β, transforming growth factor-β; IL-10, interleukin-10; FoxP3, forkhead box P3; Treg, regulatory T cell.

In this study, we first built the mouse model of EAP by subcutaneous immunity twice (**Figure 1A**). The EAP model was assessed by the result of histological examination and pelvic pain measurement. As shown in **Figures 1B–D**, respectively, compared with the control group, both prostatic inflammation and response frequency to tactile allodynia were obviously increased in the EAP group, indicating a successful establishment of the mouse EAP model. Next, we analyzed the percentage of Th17 and Treg cells in the splenic lymphocytes from the control and EAP mice. As a result, both the proportion of CD4^+^IL17^+^ cells (0.90% ± 0.11% vs. 1.18% ± 0.11%, **Figures 1E**, , *p* < 0.01) and CD4^+^RORγt^+^ cells (0.96% ± 0.18% vs. 1.47% ± 0.26%, **Figures 1G, H**, *p* < 0.05) in the EAP group were tremendously higher than that in the control group, while the frequency of CD4^+^CD25^+^FoxP3^+^ cells was significantly lower (6.42% ± 0.95% vs. 4.33% ± 0.61%, **Figures 1I, J**, *p* < 0.01), indicating a prominent imbalance of Th17/Treg in the peripheral spleen of EAP mice. We also examined the mRNA expressions of T-bet and GATA3, two transcription factors of other T-cell subsets, in the splenic lymphocytes. The results suggested that the T-bet level was obviously increased in the EAP group as compared to the control group (**Supplementary Figure 1A**, *p* < 0.05), while no statistical differences in GATA3 expression were found between the two groups (**Supplementary Figure 1B**, *p* > 0.05). Moreover, compared with control individuals, the concentrations of serum anti-inflammatory cytokines TGF-β and IL-10 were significantly decreased, while the levels of serum pro-inflammatory cytokines IL-17, GM-CSF, and IFNγ were significantly increased in the EAP group (**Figure 1K**, *p* < 0.01 or *p* < 0.05). Consistent with these results, EAP mice also have a higher IL-17 level and lower levels of TGF-β and IL-10 in prostate tissues (**Figure 1L**, *p* < 0.01 or *p* < 0.05). Lastly, we performed immunohistochemical staining of IL-17A and FoxP3 on prostate sections from the two groups. The results suggested that the proportions of IL-17A-positive staining cells were increased and the proportions of FoxP3-positive staining cells were decreased in the prostate tissues of EAP mice (**Figures 1M, N**). All these results suggested an imbalance state of Th17/Treg in EAP.

### Alterations of the Fecal Metabolome in EAP

**Figure 2:**
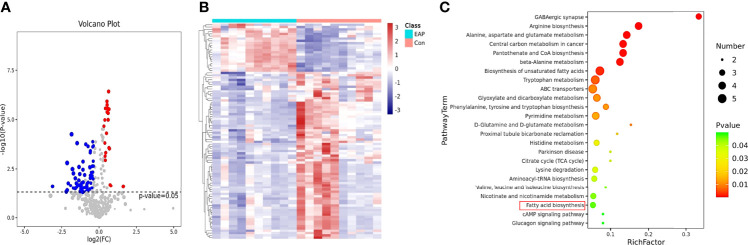
Metabolomic profiles of control and EAP mice. **(A)** Volcano plot for metabolite analysis in feces from control and EAP mice. The red dots denote the significantly upregulated metabolites, the blue dots denote the significantly downregulated metabolites, and the gray dots denote the insignificant metabolites. **(B)** A hierarchical clustering heatmap of metabolites in feces of control and EAP mice. Red represents increased expression, while green represents decreased expression. **(C)** A bubble chart of KEGG pathway enrichment. The disturbances in fatty acids are highlighted in red. *n* = 10/group; EAP, experimental autoimmune prostatitis.

The mammalian immune system is in a constant dialogue with the microbiome (25). As previously described, gut microflora plays a pivotal role in immune homeostasis and usually regulates the host immune systems by its metabolites (19). Thus, metabolomic analysis was conducted to investigate the enteric metabolic profiles of EAP mice. After untargeting metabolomics sequencing of feces by GC-MS, it was identified that a total of 94 metabolites differed in abundance between control and EAP mice (**Figures 2A, B**). Further KEGG pathway enrichment analysis of differentially expressed pathways between the control and EAP group was carried out (**Figure 2C**). Importantly, among these enriched pathways, we paid more attention to fatty acid biosynthesis due to the deep connections between fatty acid and immune cell regulation (26). Given that we had found the proportion imbalance of Th17/Treg cells in EAP previously, it was reasonable to doubt preliminarily whether fatty acids may exert an immunoregulatory effect on this process. Taken together, these data showed significant differences in gut microbiota-derived metabolites of control and EAP mice, and provided us a clue to investigate the role of fatty acids of microbiota-derived metabolites in EAP.

### Gut Microflora of EAP Significantly Differs From That of Controls

**Figure 3:**
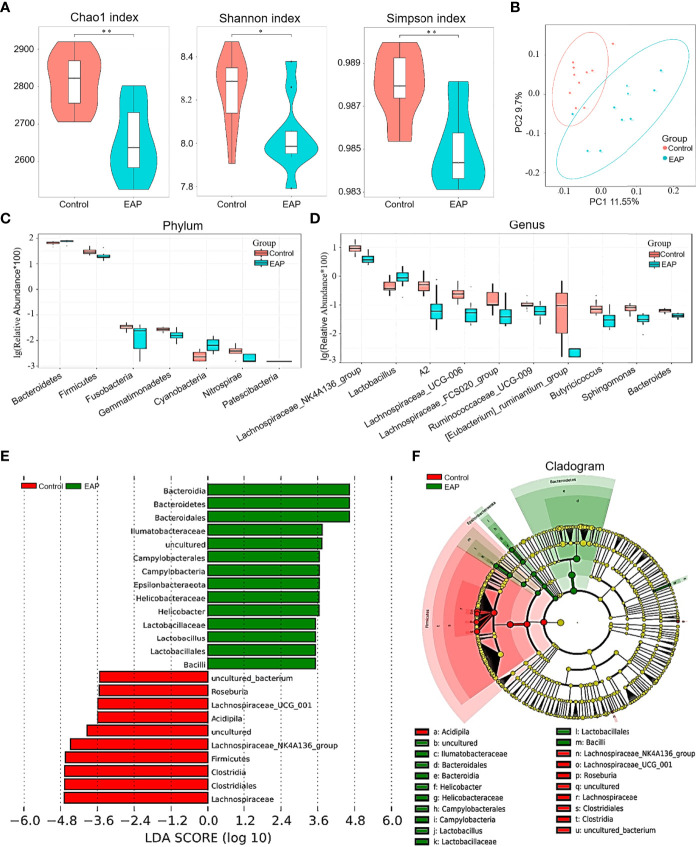
Comparison of gut microbiome between control and EAP mice. **(A)** α-diversity indices (Chao 1 index, Shannon index, and Simpson index) of bacterial 16S rRNA gene of fecal samples from control and EAP mice. **(B)** β-diversity (Bray–Curtis similarity index) of bacterial 16S rRNA genes of fecal samples from control and EAP mice. Most of the dots in the EAP group were separated from those in the control group. **(C)** Relative abundance of gut microflora at the genus level from control and EAP mice. **(D)** Relative abundance of gut microflora at the genus level from control and EAP mice. **(E)** A LEfSe analysis of differentially abundant genera from control and EAP mice. **(F)** A cladogram of the differentially abundant bacteria from control and EAP mice. *n* = 10/group; *p < 0.05; **p < 0.01; EAP, experimental autoimmune prostatitis.

To determine the role of gut microflora in EAP, 16S ribosomal RNA sequencing was performed on fecal samples. A Venn diagram showed the number of common and unique OTUs in the control and EAP groups (**Supplementary Figure 2A**). The parameters of α-diversity indices, including Chao1, Shannon index, Simpson index, observed_species, and PD_whole_tree, were all markedly lower in EAP mice than those in the controls, revealing a significantly altered α-diversity of the gut microbiota (**Figure 3A** and **Supplementary Figure 2B**, *p* < 0.05 or *p* < 0.01). The PCoA analysis, an indicator of β-diversity, showed a noticeable separation between the samples of control and EAP mice, suggesting significantly different microbiota composition between the two groups (**Figure 3B**). At the phylum level, EAP mice had fewer relative abundance of Firmicutes, Nitrospirae, Gemmatimonadetes, and Fusobacteria. Conversely, we detected an increase in Bacteroidetes, Cyanobacteria, and Patescibacteria in the EAP group (**Figure 3C**, *p* < 0.05). At the genus level, *Lactobacillus* was markedly increased in EAP, while the other nine flora were obviously decreased (**Figure 3D**, *p* < 0.05). Notably, several members of these decreased bacterial genus, including *Bacteroides*, *Butyricicoccus*, and *Ruminococcaceae_UCG_009*, were the so-called “SCFAs-producing bacteria” that metabolize indigestible carbohydrates to immunogenic SCFAs as reported in the literature (27–29). The relative abundance of gut microbiota composition in the EAP group at the class level, order level, family level, and species level was also significantly altered in EAP mice in comparison with the controls (**Supplementary Figures 2C–F**). Next, a linear discriminant analysis effect size (LEfSe) analysis was performed to explore the bacteria with higher abundance in the two groups, which has the potential to serve as biomarkers. Overall, 24 genera were determined with LDA scores >3.5 (**Figure 3E**). A cladogram of annotated branches of different bacteria is shown in **Figure 3F**. Intriguingly, phylogenetic investigation of communities by reconstruction of unobserved states (PICRUSt) analysis predicted multiple metabolic pathways including SCFA metabolism (butanoate metabolism and propanoate metabolism) that might be highly relevant to EAP (**Supplementary Figure 3**).

### The SCFA Propionate Decreases Significantly in EAP

**Figure 4:**
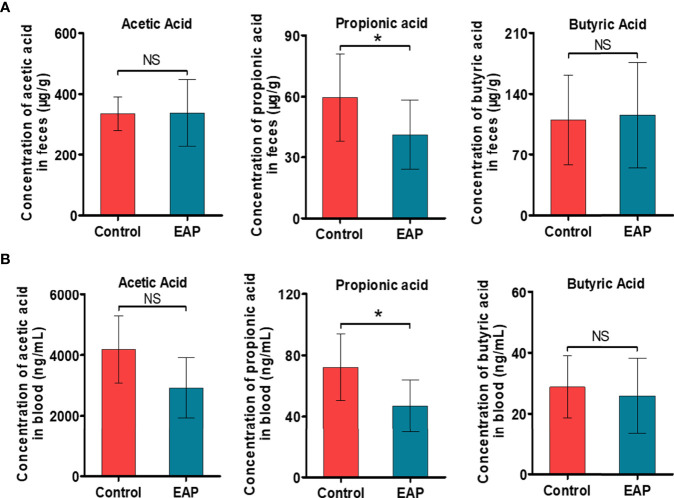
Detection of the absolute concentrations of SCFAs, including acetic acid, propionic acid, and butyric acid, in control and EAP mice. **(A)** The absolute concentrations of several common SCFAs (acetic acid, propionic acid, and butyric acid) in feces from control and EAP mice. **(B)** The absolute concentrations of acetic acid, propionic acid, and butyric acid in serum from control and EAP mice. Data are representative of three independent experiments. *n* = 6–10/group; *p < 0.05; NS, not significant; EAP, experimental autoimmune prostatitis; SCFAs, short-chain fatty acids.

Building on the above findings in KEGG pathway enrichment analysis of untargeted metabolomics and PICRUSt analysis of 16S rRNA sequencing, we are next committed to investigating the alterations of fatty acids in EAP, especially the SCFAs. The concentrations of the six most abundant SCFAs, namely, acetic acid, propionic acid, butyric acid, pentanoic acid, isovaleric acid, and isobutyric acid, in both feces and serum were measured. As a result, the concentrations of propionic acid in mice feces were higher in the control groups than those in the EAP group (**Figure 4A**, *p* < 0.05), but no differences existed in other SCFAs between the two groups (**Figure 4A** and **Supplementary Figure 4A**, *p* > 0.05). Analysis of SCFA concentrations in the serum of EAP mice also presented a striking decrease in content of propionic acid (**Figure 4B**, *p* < 0.05) but not obviously in other acids of SCFAs as compared to controls (**Figure 4B** and **Supplementary Figure 4B**, *p* > 0.05). These data demonstrated that the microbiota-derived propionic acid was significantly decreased in EAP.

### Propionic Acid Supplementation Ameliorates EAP and Restores Th17/Treg Imbalance *In Vivo*

**Figure 5:**
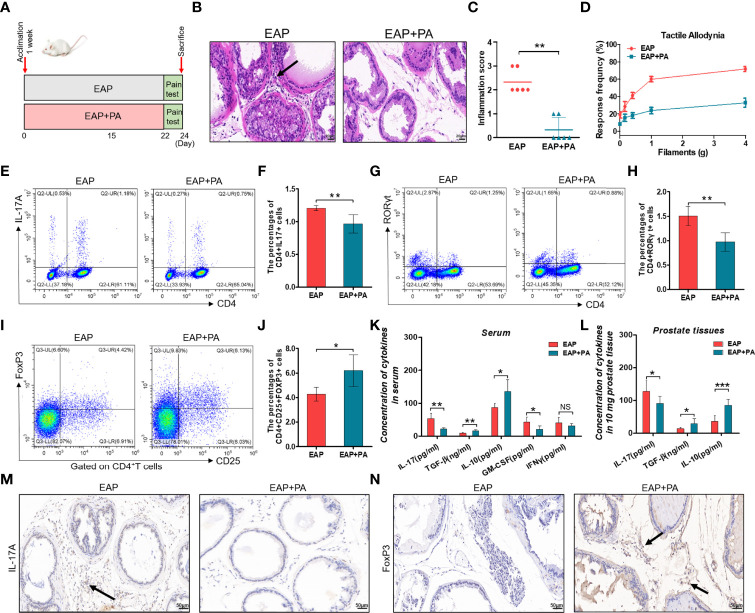
Propionic acid ameliorates EAP and restores Th17/Treg imbalance *in vivo.*** (A)** After 1 week of acclimation, all mice received EAP induction. They were divided into an untreated group (EAP group) and a propionate-treated group (EAP+PA group) according to whether propionic acid was added in drinking water or not. Mice were sacrificed on day 24 and then samples were collected. **(B)** Representative HE staining of prostate sections from untreated and propionate-treated EAP mice. The black arrowhead indicates infiltration of inflammatory cells in the EAP group. **(C)** Inflammation score of prostate sections from untreated and propionate-treated EAP mice. **(D)** Chronic pelvic pain measurement of untreated and propionate-treated EAP mice. **(E)** Representative pictures of flow cytometric staining for CD4^+^IL-17^+^ cells in the splenic lymphocytes from untreated and propionate-treated EAP mice. **(F)** Flow cytometric analysis of the proportion of CD4^+^IL-17^+^ cells in untreated and propionate-treated EAP mice. **(G)** Representative pictures of flow cytometric staining for CD4^+^RORγt^+^ cells in the splenic lymphocytes from untreated and propionate-treated EAP mice. **(H)** Flow cytometric analysis of the proportion of CD4^+^RORγt^+^ cells in untreated and propionate-treated EAP mice. **(I)** Representative pictures of flow cytometric staining for CD4^+^CD25^+^FoxP3^+^ cells in the splenic lymphocytes from untreated and propionate-treated EAP mice. **(J)** Flow cytometric analysis of the proportion of CD4^+^CD25^+^FoxP3^+^ cells in untreated and propionate-treated EAP mice. **(K)** Detection of serum cytokine productions (IL-17, GM-CSF, IFNγ, TGF-β, and IL-10) from untreated and propionate-treated EAP mice. **(L)** Concentration determination of IL-17, TGF-β, and IL-10 in 10 mg of prostate tissue in untreated and propionate-treated EAP mice. **(M)** Immunohistochemical staining of IL-17A (marker of Th17) on prostate sections in untreated and propionate-treated EAP mice. **(N)** Immunohistochemical staining of FoxP3 (marker of Treg) on prostate sections in untreated and propionate-treated EAP mice. The black arrows indicate the IL-17/FoxP3-positive cells in the two groups. Data are from one experiment representative of three independent experiments. Data are representative of three independent experiments. *n* = 5–6/group; *p < 0.05; **p < 0.01; ***p < 0.001; NS, not significant; EAP, experimental autoimmune prostatitis; HE, hematoxylin–eosin, PA, propionic acid; IL-17, interleukin-17; RORγt, retinoic acid−related orphan receptor γt; GM-CSF, granulocyte-macrophage colony stimulating factor; IFNγ, interferon gamma; TGF-β, transforming growth factor-β; IL-10, interleukin-10; FoxP3, forkhead box P3; Treg, regulatory T cell.

To elucidate the impacts of propionic acid on EAP, we first utilized it *in vivo* experiments. EAP mice were divided into an untreated group (EAP group) and a propionate-treated group (EAP+PA group) according to whether propionic acid was added in drinking water or not (**Figures 5A**). Notably, supplementation with exogenous propionic acid significantly reduced the prostatic inflammation and pelvic pain of EAP mice (**Figures 5B–D**). Furthermore, propionic acid treatment suppressed Th17 cell differentiation in the splenic lymphocytes of EAP mice (**Figures 5E–H**, *p* < 0.05) but favored Treg cell differentiation (**Figures 5I, J**, *p* < 0.05). In addition, oral supplementation with propionic acid decreased the production of IL-17 and GM-CSF in serum of EAP mice, while it increased the secretion of TGF-β and IL-10 (**Figure 5K**, *p* < 0.05 or *p* < 0.01). Similar results to the levels of IL-17, TGF-β, and IL-10 were observed in prostate tissues (**Figure 5L**, *p* < 0.05 or *p* < 0.001). Additionally, we also performed immunohistochemical staining of IL-17A and FoxP3 on prostate sections from the two groups (**Figures 5M, N**). The results indicated that the proportion of IL-17A-positive staining cells were decreased while the proportion of FoxP3-positive staining cells were increased in the prostate tissues of propionate-treated EAP mice. Collectively, these observations suggested that propionic acid helped to ameliorate the prostatic inflammation and restore Th17/Treg imbalance induced by EAP.

### Propionic Acid Regulates Th17/Treg Cell Differentiation *In Vitro*

**Figure 6:**
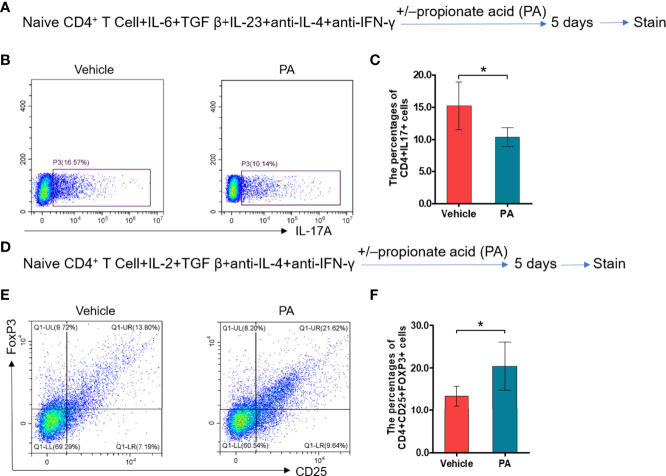
Propionic acid regulates Th17/Treg cell differentiation *in vitro.*** (A)** Sorted naive CD4^+^ T cells were activated for 5 days under Th17 cell differentiation conditions with or without propionic acid and subjected to flow cytometry analysis for IL-17a. **(B)** Representative pictures of flow cytometric staining for CD4^+^IL-17^+^ cells from the vehicle group (without propionic acid treatment) and the PA group (incubated with propionic acid). **(C)** Flow cytometric analysis of the CD4^+^IL-17^+^ cells’ proportion. **(D)** Sorted naive CD4^+^ T cells were activated for 5 days under Treg cell differentiation conditions with or without propionic acid and subjected to flow cytometric analysis for FoxP3. **(E)** Representative pictures of flow cytometric staining of CD4^+^CD25^+^FoxP3^+^ cells from the vehicle group and the PA group. **(F)** Flow cytometric analysis of the CD4^+^CD25^+^FoxP3^+^ cell proportion. Data are representative of three independent experiments. *n* = 5/group; *p < 0.05; PA, propionic acid; IL-17, interleukin-17; FoxP3, forkhead box P3; Treg, regulatory T cell.

We further assess the impacts of propionic acid on EAP by performing *in vitro* experiments. Propionic acid was administered to isolate CD4^+^ naive T cells that were under Th17/Treg differentiation conditions (**Figures 6A, D**). As expected, propionic acid could suppress more CD4^+^ naive T cells to differentiate into Th17 cells (**Figures 6B, C**, *p* < 0.05), while it was also associated with a promotion for the differentiation into Treg cells (**Figures 6E, F**, *p* < 0.05). These data demonstrated that propionic acid helped to regulate Th17/Treg balance *in vitro*, further strengthening the immune-modulatory role of propionic acid in EAP.

### Propionic Acid Regulates Th17/Treg Cell Differentiation by Modulation of the GPR43–HDAC6 Axis

**Figure 7:**
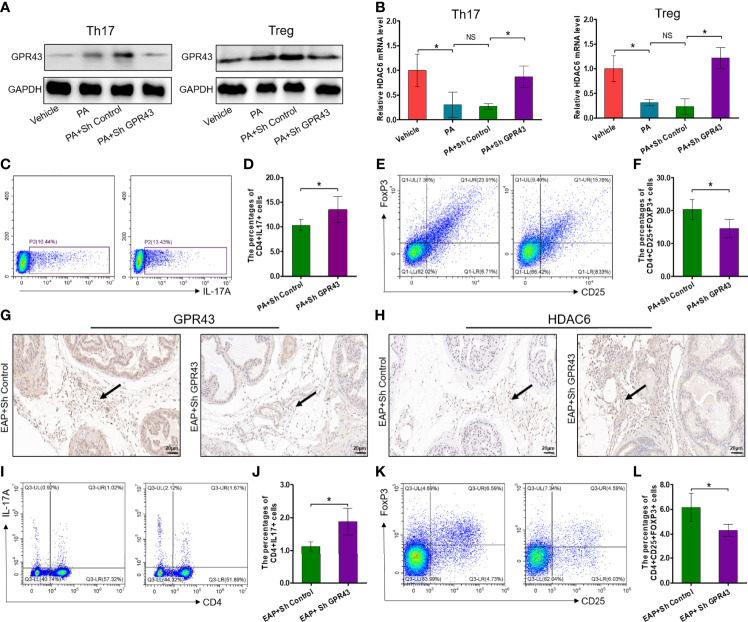
Propionic acid regulates Th17/Treg cell differentiation by modulation of the GPR43–HDAC6 axis. **(A)** Immunoblot analysis of GPR43 expression levels in CD4^+^ T cell under Th17/Treg differentiation conditions that were assigned to four groups: vehicle group (without treatment), PA group (incubated with propionic acid), PA+sh Control group (incubated with propionic acid and LV-sh Control), and PA+sh GPR43 group (incubated with propionic acid and LV-sh GPR43). **(B)** The relative mRNA expressions of HDAC6 in the four groups as detected by RT-qPCR assay. **(C)** Representative pictures of flow cytometric staining for CD4^+^IL-17^+^ cells in the PA+sh Control group and the PA+sh GPR43 group. **(D)** Flow cytometric analysis of the proportion of CD4^+^IL-17^+^ cells in the PA+sh Control group and the PA+sh GPR43 group. **(E)** Representative pictures of flow cytometric staining for CD4^+^CD25^+^FoxP3^+^ cells in the PA+sh Control group and the PA+sh GPR43 group. **(F)** Flow cytometric analysis of the proportion of CD4^+^CD25^+^FoxP3^+^ cells in the PA+sh Control group and the PA+sh GPR43 group. **(G)** Immunohistochemical analysis of GPR43 expression in prostate of EAP mice treated with LV-sh Control or LV-sh GPR43. Results indicate a decrease in GPR43 expression in prostatitic inflammatory cells of EAP mice treated with LV-sh GPR43. The black arrows indicate the GPR43-positive inflammatory cells in the two groups. **(H)** Immunohistochemical analysis of HDAC6 expression in prostate of EAP mice treated with LV-sh Control or LV-sh GPR43. Results indicate an increase in HDAC6 expression in prostatitic inflammatory cells of EAP mice treated with LV-sh GPR43. The black arrows indicate the HDAC6-positive inflammatory cells in the two groups. **(I)** Representative pictures of flow cytometric staining for CD4^+^IL-17^+^ cells in EAP mice treated with LV-sh Control or LV-sh GPR43. **(J)** Flow cytometric analysis of the proportion of CD4^+^IL-17^+^ cells in in EAP mice treated with LV-sh Control or LV-sh GPR43. **(K)** Representative pictures of flow cytometric staining for CD4^+^CD25^+^FoxP3^+^ cells in the splenic lymphocytes from EAP mice treated with LV-sh Control or LV-sh GPR43. **(L)** Flow cytometric analysis of the proportion of CD4^+^CD25^+^FoxP3^+^ cells in EAP mice treated with LV-sh Control or LV-sh GPR43. Data are representative of three independent experiments. *n* = 3–5/group; *p < 0.05; **p < 0.01; EAP, experimental autoimmune prostatitis; SCFA, short-chain fatty acid; NS, not significant; PA, propionic acid; GPR43, G-protein-coupled receptor 43; HDAC6, histone deacetylase 6; LV, lentivirus; IL-17, interleukin-17; FoxP3, forkhead box P3; Treg, regulatory T cell.

It has been exquisitely reported that SCFA propionate could regulate immune cell differentiation through G-protein-coupled receptors (GPRs) and histone deacetylases (HDACs) (30). To explore whether propionic acid regulated Th17 and Treg cell differentiation in a GPR-dependent manner, we detected the expressions of GPR43, one of the GPRs that play an important role in the immunity and metabolism, and intracellular signaling HDAC6 (31). First, the expressions of GPR43 were increased by propionic acid stimulation under Th17 and Treg cell conditions but suppressed by using LV-shGPR43 (**Figure 7A**). We also observed that when isolated CD4^+^ T cells were treated with propionic acid and incubated under Th17/Treg differentiation conditions, the expression level of HDAC6 was decreased. However, after LV-shGPR43 infection, it was increased significantly (**Figure 7B**, *p* < 0.05). Additionally, the suppression of GPR43 contributed to a sharp increase in Th17 cell differentiation (**Figures 7C, D**, *p* < 0.05) and an obvious reduction in Treg cell differentiation (**Figures 7E, F**, *p* < 0.05), which reversed the effects of propionic acid on Th17/Treg differentiation. In subsequent animal experiments, we also used LV-mediated RNA interference to knock down GPR43 in EAP mice (suppression of GPR43 was confirmed in **Supplementary Figures 5A, B**). The immunohistochemical detection of prostate tissue exhibited a reduction of GPR43-positive cells and an increment of the expression levels of HDAC6 in EAP mice treated with LV-sh GPR43 (**Figures 7G, H**). Knocking down GPR43 eventually led to an imbalance of Th17/Treg in these mice (**Figures 7I–L**, *p* < 0.05). On the basis of the above data, we proposed that SCFA propionate may regulate Th17/Treg cell differentiation by the GPR43–HDAC6 axis.

### Fecal Transplantation From EAP Induces the Decrease of Treg Cell Frequency in Recipient

**Figure 8:**
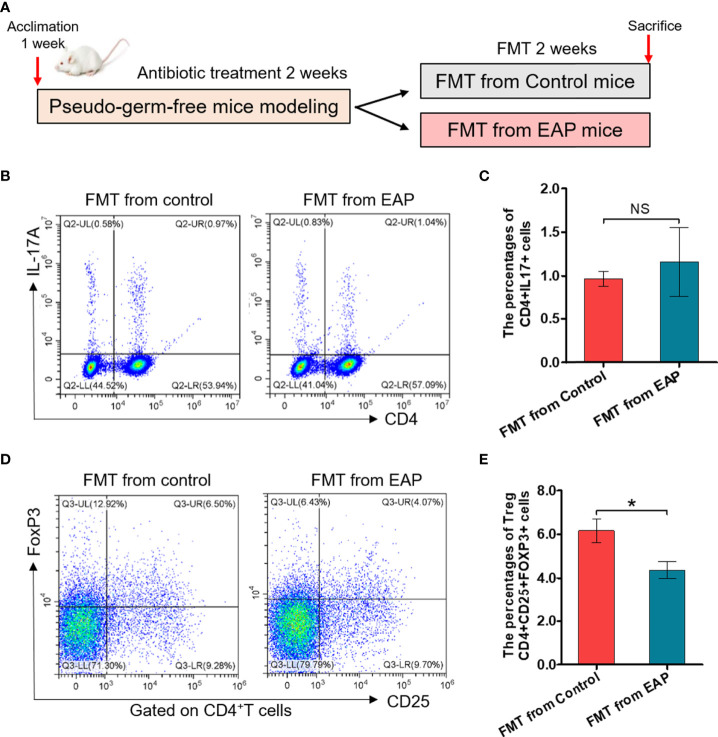
Fecal transplantation from EAP mice induces the decrease of Treg cell frequency in recipient mice. **(A)** Sketch of the fecal transplantation process. After 1 week of acclimation, mice were treated with antibiotics for 2 weeks. Subsequently, mice received 2 weeks of fecal transplantation from control and EAP mice. All mice were sacrificed on day 28, and samples were collected. **(B)** Representative pictures of flow cytometric staining for CD4^+^IL-17^+^ cells in the splenic lymphocytes of mice that respectively received fecal microbiota transplantation from control and EAP mice. **(C)** Flow cytometric analysis of the proportion of CD4^+^IL-17^+^ cells in recipient mice that respectively received fecal microbiota transplantation from control and EAP mice. **(D)** Representative pictures of flow cytometric staining for CD4^+^CD25^+^FoxP3^+^ cells in the splenic lymphocytes of mice that respectively received fecal microbiota transplantation from control and EAP mice. **(E)** Flow cytometric analysis of the proportion of CD4^+^CD25^+^FoxP3^+^ cells in recipient mice that respectively received fecal microbiota transplantation from control and EAP mice. Data are representative of three independent experiments. *n* = 4–5/group; *p < 0.05; NS, not significant; EAP, experimental autoimmune prostatitis; FMT, fecal microbiota transplantation; IL-17, interleukin-17; FoxP3, forkhead box P3; Treg, regulatory T cell.

To further determine whether modifications of the gut microbiota were responsible for imbalance of Th17/Treg in EAP, we took advantage of feces transplantation experiments by gavaging feces suspensions from control or EAP mice into antibiotic-treated pseudo-germ-free recipient mice (**Figure 8A**). As shown in **Figures 8B–E**, the proportion of Treg cells in fecal transplantation from the EAP mice was markedly decreased compared to fecal transplantation from the control mice (*p* < 0.05), although no statistical differences existed in the proportion of Th17 cells between the two groups (*p* > 0.05). Thus, the oral transfer of intestinal microbes from EAP donors into healthy recipients induced their decrease of Treg cell frequency.

## Discussion

CP/CPPS remains one of the leading diseases endangering men’s health, especially for young men. Whereas more is understood regarding the participation of Th17/Treg cells in CP/CPPS, the regulatory mechanism of Th17/Treg cell differentiation remains underexplored. Recent breakthroughs in our understanding of the important role of the gut microflora in immune cells have novel implications for investigating the pathogenesis of CP/CPPS (32). In this research, we showed changes in the diversity and relative abundance of gut microflora in response to EAP resulting in extensive alterations in the host metabolites, and also observed that the fatty acid-related pathway was enriched in the EAP group. Further probing revealed that EAP mice have obviously decreased contents of SCFA propionate in both feces and serum, and propionic acid supplementation attenuated EAP severity and restored its Th17/Treg balance, which was possibly linked to the GPR43 activation and HDAC6 inhibition. Our research broadens the horizons of the correlation between gut microflora and extra-intestinal organs or immune cells and provided new lines of evidence for the previously proposed concept of the “gut–prostate” axis (33).

It has been now becoming widely accepted that the large amounts of commensal gut bacteria not only are restricted to regulate the intestinal mucosal immunity and maintain the intestinal homeostasis, but also exert systemic effects on immune cells in extra-intestinal organs or tissues (34). The generative mechanism for these influences on distal anatomical sites, however, needs continued research. In the prostate, several studies have shown that gut dysbiosis can induce a chronic inflammatory state and disturb systemic hormonal production, which may be the potential causes of prostate cancer (35, 36). Nonetheless, few studies have explored the mechanistic basis of the occurrence of prostatitis in response to gut dysbiosis. Shoskes et al. (16) first found that patients with CPPS have markedly less gut microbiome diversity in clusters that is different from controls. Then, Liu et al. (33) took a big step forward by suggesting an epigenetic mechanism for chronic nonbacterial prostatitis that is correlated with gut microbiota, immunity, and metabolism using multi-omics analysis. Since Th17 and Treg cells are the key immune cells that may regulate the development of CP/CPPS under the condition of when Th17 cells are activated and Treg cells are inhibited (37), it is necessary to demonstrate the gut dysbiosis and Th17/Treg cell immune imbalance in EAP and uncover whether there was an objective arrived-at connection between them. Here, we first investigated the immune state of Th17 and Treg cells after EAP induction and demonstrated the imbalance of Th17/Treg in periphery immune organ spleen of EAP mice. Interestingly in our previous study, after the start of the EAP model, the proportion of Treg cells increased on day 42 in comparison with the control group (38). In this study, by contrast, the proportion of Treg cells of EAP mice decreased significantly on day 24. We reasoned that EAP mice may have different immune states at different stages of modeling. The proportion of Treg cells in the EAP model might change from a decrease to an increase with the passage of time, which was helpful to the reduction of inflammation. This is also consistent with the clinical observation that a small number of patients with CP/CPPS recover without any treatment after a period of time. Also, what we investigated then was the parameters of gut microflora and its metabolites in EAP mice by fecal 16s rRNA and cecal metabolomics sequencing. Our results implied that changes in the constitution of gut microflora resulted in profound alterations in host metabolite abundance. Previous studies have reported that the abundance of *Lactobacillus* was negatively correlated with Treg cells and IL-10 level in spleen, while the abundance of *Bacteroides* was positively related to them (39). In another study, researchers also found that a branch of *Bacteroides* could produce propionic acid and increase Treg cell numbers while decreasing Th17 cell numbers (19). Consistent with these studies, the abundance of *Bacteroides* and *Lactobacillus* was also altered in this study and may be the key genera that were associated with SCFA propionate metabolism and immune regulation. Altogether, our findings provide a promising blueprint of the correlation between gut microflora dysbiosis and immune inflammation of prostate through the communication of Th17/Treg cell differentiation imbalance.

The gut microbiota regulates the host immunity mainly by its metabolism, such as trimethylamine oxide, bile acid, and amino acids. Among these gut-microbiota-derived metabolites, SCFAs have emerged as important microbial metabolites participating in the modulation of immune inflammation and metabolism by interactions with both gut microbiome and host receptors (18). Recent literature has demonstrated that SCFAs are an effective class of immune-modulatory mediators with the capacity to regulate Th17 and Treg cell differentiation in gut and periphery (40, 41). To date, only a few studies have reported on the beneficial influences of SCFAs in the CP/CPPS mouse model. Here, based on our microbiome analysis findings of the decrease of several SCFA-producing bacteria and the prediction that SCFAs may be involved, we focused on the detection of the content of SCFAs and observed that the contents of propionic acid were markedly decreased in EAP mice, whereas other types of SCFAs, such as acetic acid and butyric acid, did not change. Importantly, the absolute concentration of propionic acid in serum was also decreased in EAP mice. It was therefore inferred that decreases in abundance of SCFA-producing bacteria may trigger decreases in SCFA propionate level and further exert effects on prostate.

Next, we further evaluated the effects of propionic acid on EAP by performing *in vivo* and *in vitro* experiments. Our work determined that SCFA propionate alleviated prostatic inflammation and pelvic pain of EAP mice. Hence, incorporating the SCFA propionate into the diet may be a promising method for treating CP/CPPS. More significantly, we noted that propionic acid supplement regulated host immune responses by diminishing the percentage of Th17 cells and restoring Treg cell proportion in EAP. The immune-regulatory effects of propionic acid were also verified in an *in vitro* study. Recent advances have identified that SCFAs can bind to GPRs, such as GPR41 and GPR43, on immune cells and further cause epigenetic alterations in the genome by changing the activity of both histone acetylase and HDAC enzymes (42, 43). Another study also reported that SCFAs, especially propionic acid, were important signaling molecules acting as GPR activators and HDAC inhibitors (44). In agreement with these studies, we found that the level of GPR43 was obviously upregulated, whereas the expression of HDAC6 mRNA was apparently downregulated in the group of SCFA propionate supplementation, which suggested that microbiota-derived propionic acid inhibited HDAC6 possibly through activating GPR43 and finally regulated Treg and Th17 cells in EAP. Furthermore, knocking down GPR43 can reverse this result and drive up rates of Th17/Treg. Hereby, the mechanisms on how propionic acid regulated the host Th17/Treg cell differentiation in EAP are probably attributed to the modulation of the GPR43–HDAC6 axis.

Nowadays, fecal transplantation has been an effective method for exploring the relationship between specific diseases and gut microflora and is a promising treatment option for several autoimmune diseases. Our data demonstrated that fecal transplantation from EAP mice resulted in a decrease in the Treg cell frequency of recipient mice. It is notable, however, that the fecal microbiota transplants from EAP mice were not sufficient to induce Th17 cell immunoreactivity in healthy recipients, suggesting an intricate relationship between gut microbiota and Th17/Treg differentiation of EAP. Previous research has indicated that loss of gut barrier integrity might be a causal factor on inducing a certain T-cell activation and autoimmune disease (45). Considering that there is no loss of gut barrier integrity in recipient mice, modifications of the gut microbiota by fecal transplantation may not fully lead to develop immune imbalance in EAP. Another possible explanation for our data may be that the fecal transplantation results in incomplete colonization of the recipient with all donor bacterial species. In addition, unlike direct SCFA administration, the effect of fecal transplantation on recipient mice is difficult to judge due to the complexity of mouse fecal composition, which warrants further research.

There are several limitations to the present study. First, although the immune state of Th17/Treg in EAP was investigated mainly by using the most generally used immune organ, spleen, it remains to further determine whether other immune organs or tissues, such as draining lymph nodes, have immune changes similar to spleen. Second, when detecting the expressions of HDAC6 in Th17 and Treg cell differentiation conditions, we did not sort Th17 cells from Treg cells to determine the HDAC6 levels in particular to each cell type.

In conclusion, our research supported preliminary evidence that the immune imbalance of Th17/Treg in EAP is mediated systemically by SCFA propionate derived from the gut microbiota. This study also provided a new perspective for improving immunological disturbance in CP/CPPS patients by supplementation of microbial end-products during dietary interventions.

## Data Availability Statement

The datasets presented in this study can be found in online repositories. The name of the repository and accession number(s) can be found below: NCBI Sequence Read Archive; accession number PRJNA827477.

## Ethics Statement

The animal study was reviewed and approved by The Institutional Animal Care and Use Committee of Anhui Medical University.

## Author Contributions

ChuL, XGC, LZ, and CZL conceived and designed the experiments. HXD, SYY, and DN performed the experiments and analyzed the data. LGZ, YC, and JC helped with the animal experiments. YG, XLH, and ChaL helped with the *in vitro* experiments. HXD, SYY, and DN wrote the manuscript. ChaL, XGC, LZ, and CZL checked the manuscripts. All authors contributed to the article and approved the submitted version.

## Funding

This work was supported by the Key Project of National Natural Science Foundation of China (81630019), the National Natural Science Fund of China (82100815, 82170787 and 81870519), and the Anhui Natural Science Foundation (2108085QH315).

## Conflict of Interest

The authors declare that the research was conducted in the absence of any commercial or financial relationships that could be construed as a potential conflict of interest.

## Publisher’s Note

All claims expressed in this article are solely those of the authors and do not necessarily represent those of their affiliated organizations, or those of the publisher, the editors and the reviewers. Any product that may be evaluated in this article, or claim that may be made by its manufacturer, is not guaranteed or endorsed by the publisher.

## Supplementary Material

Supplementary Figure 1 The relative mRNA expressions of transcription factor T-bet and GATA3 in the splenic lymphocytes from control and EAP mice. **(A)** The relative mRNA expression of transcription factor T-bet in the splenic lymphocytes from control and EAP mice. **(B)** The relative mRNA expression of transcription factor GATA3 in the splenic lymphocytes from control and EAP mice. Data are the representative of three independent experiments. n=3/group; *, *P*<0.05; NS, not significant; EAP, experimental autoimmune prostatitis; T-bet, T-box expressed in T cells; GATA3, GATA binding protein 3.

Supplementary Figure 2 Comparison of gut microbiome between control and EAP mice. **(A)** A Venn diagram of OTUs in EAP and control mice. **(B)** α-diversity indices (observed_species, and PD_whole_tree) of bacterial 16S rRNA gene of fecal samples from control and EAP mice. **(C)** Relative abundance of gut microflora at class, order, family, and species levels from control and EAP mice. n=10/group; **, *P*<0.01; EAP, experimental autoimmune prostatitis; OTUs, operational taxonomic units.

Supplementary Figure 3 PICRUSt analysis revealed that the metabolic pathways of butanoate metabolism and propanoate metabolism (highlighted in red boxes) might be relevant to EAP. n=10/group; EAP, experimental autoimmune prostatitis; PICRUSt, phylogenetic investigation of communities by reconstruction of unobserved states.

Supplementary Figure 4 Detection of the absolute concentrations of SCFAs, including pentanoic acid, isobutyric acid, and isovaleric acid, in control and EAP mice **(A)** The absolute concentrations of SCFAs, including pentanoic acid, isobutyric acid, and isovaleric acid, in feces from control and EAP mice. **(B)** The absolute concentrations of pentanoic acid, isobutyric acid, and isovaleric acid in serum from control and EAP mice. Data are the representative of three independent experiments. n=6-10/group; NS, not significant; EAP, experimental autoimmune prostatitis; SCFAs, short-chain fatty acids.

Supplementary Figure 5 Analysis of the GPR43 protein expression levels and the relative mRNA expressions of HDAC6 in different groups. **(A)** Immunoblot analysis of GPR43 expression levels in Treg cells that were isolated from mice of four groups: EAP group, EAP+PA group, EAP+PA+sh Control group, and EAP+PA+sh GPR43 group. **(B)** The relative mRNA expressions of HDAC6 in Treg cells that were isolated from mice of the four groups. n=3/group; *, *P*<0.05; NS, not significant; EAP, experimental autoimmune prostatitis; PA, propionic acid; GPR43, G-protein coupled receptors 43; HDAC6, histone deacetylase 6; Treg, regulatory T cell.
