# Supplementary figures and images for: Gut Microflora Modulates Th17/Treg Cell Differentiation in Experimental Autoimmune Prostatitis via the Short-Chain Fatty Acid Propionate
Source: Front Immunol. 2022 Jul 4;13:915218. doi: 10.3389/fimmu.2022.915218 (PMC9289123; doi:10.3389/fimmu.2022.915218)

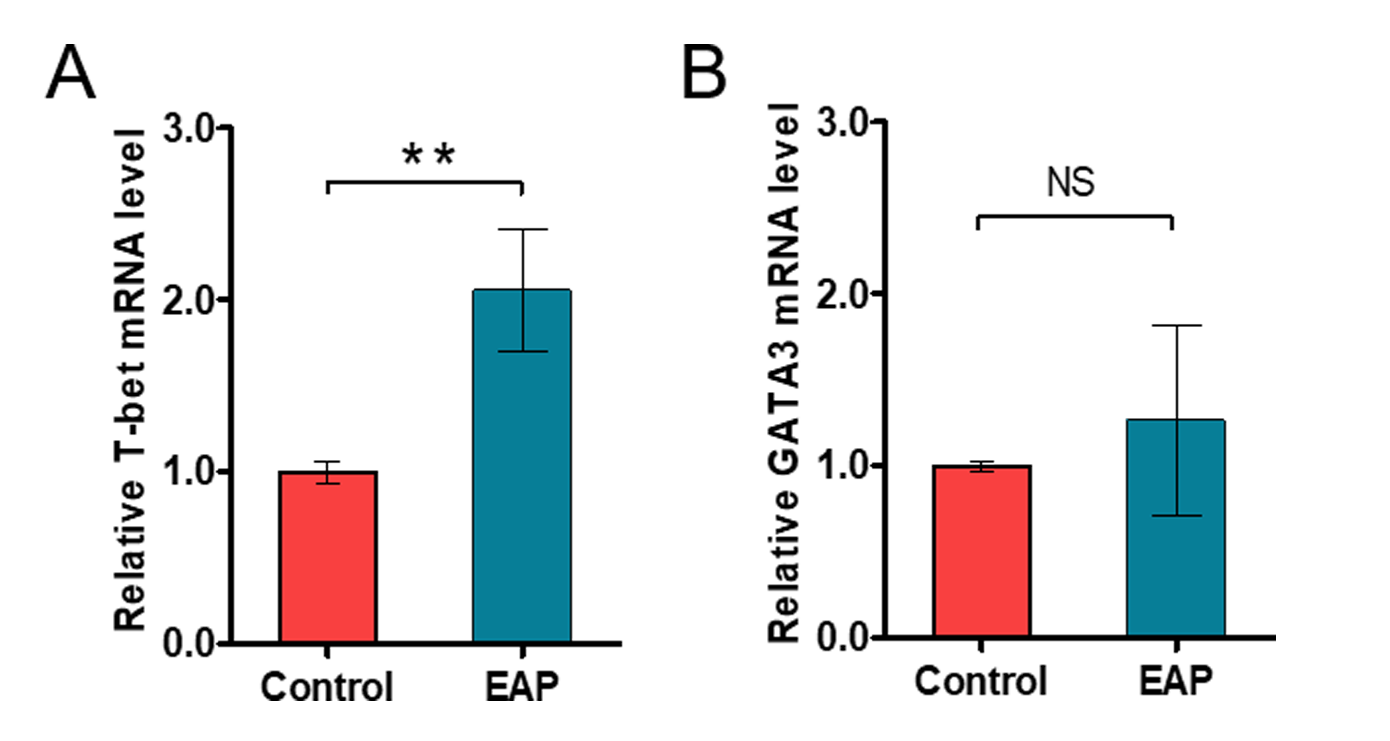

Supplement: Supplementary Figure 1 — The relative mRNA expressions of transcription factor T-bet and GATA3 in the splenic lymphocytes from control and EAP mice. (A) The relative mRNA expression of transcription factor T-bet in the splenic lymphocytes from control and EAP mice. (B) The relative mRNA expression of transcription factor GATA3 in the splenic lymphocytes from control and EAP mice. Data are the representative of three independent experiments. n=3/group; *, P<0.05; NS, not significant; EAP, experimental autoimmune prostatitis; T-bet, T-box expressed in T cells; GATA3, GATA binding protein 3. [file Image_1.tif]

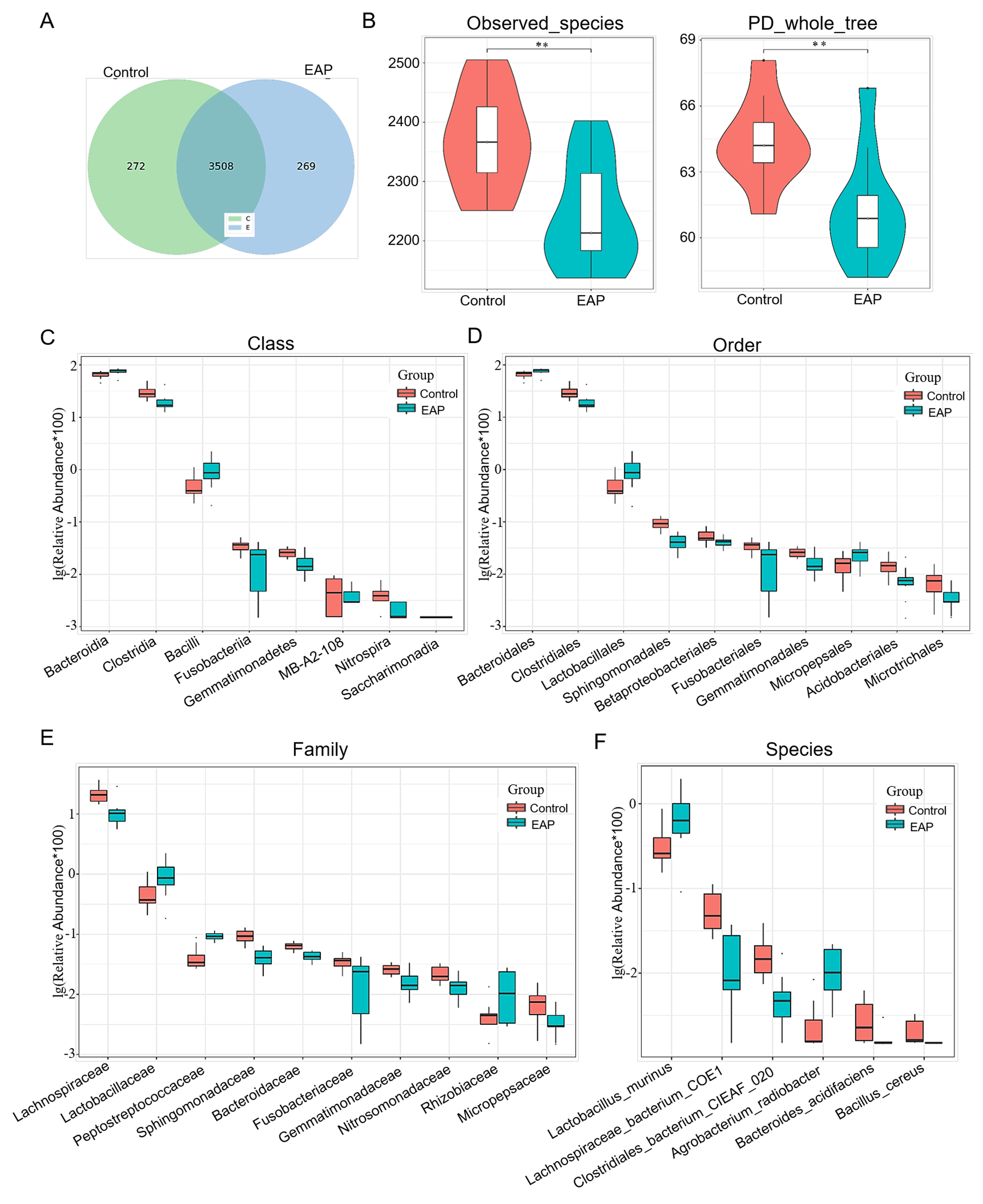

Supplement: Supplementary Figure 2 — Comparison of gut microbiome between control and EAP mice. (A) A Venn diagram of OTUs in EAP and control mice. (B) α-diversity indices (observed_species, and PD_whole_tree) of bacterial 16S rRNA gene of fecal samples from control and EAP mice. (C) Relative abundance of gut microflora at class, order, family, and species levels from control and EAP mice. n=10/group; **, P<0.01; EAP, experimental autoimmune prostatitis; OTUs, operational taxonomic units. [file Image_2.tif]

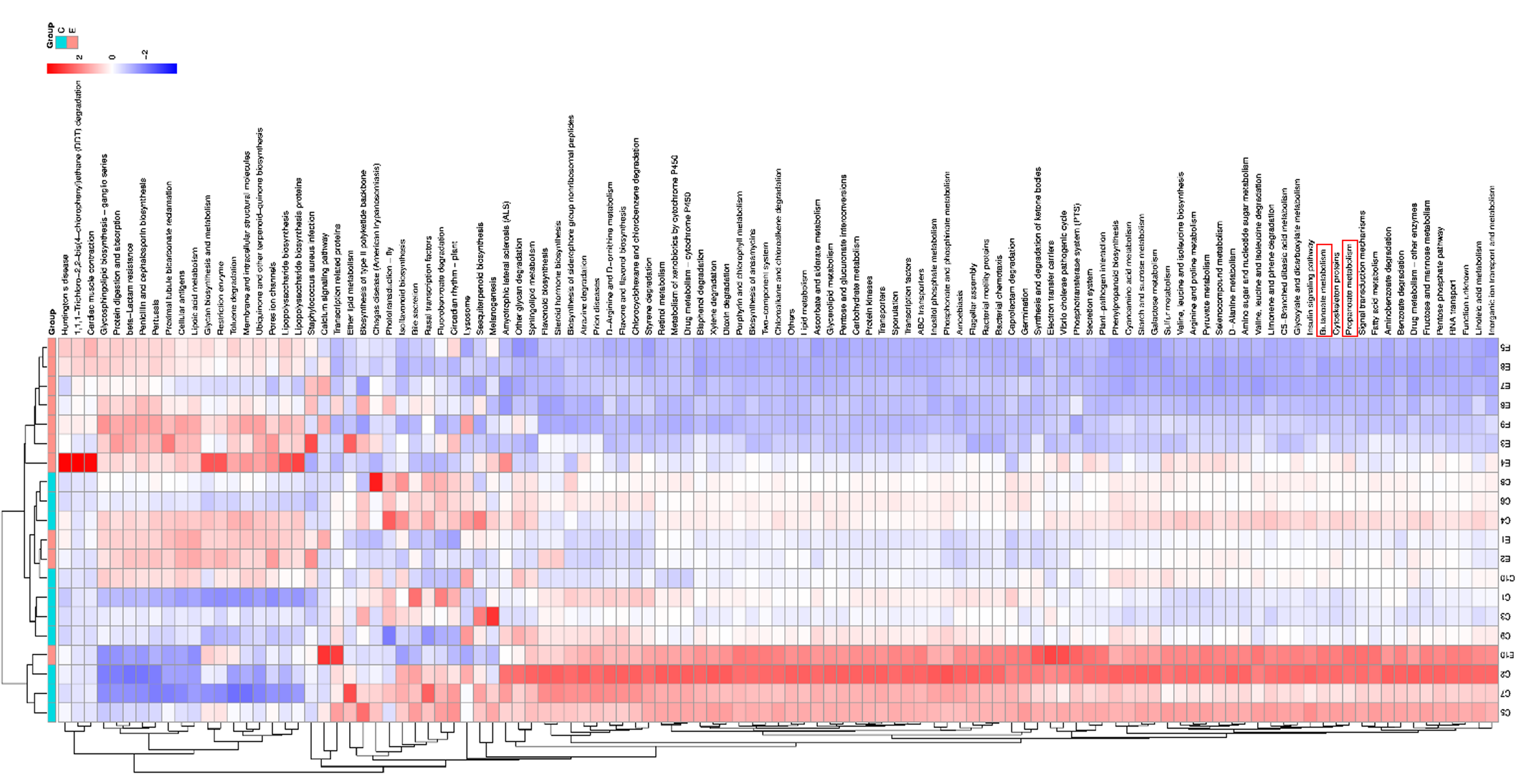

Supplement: Supplementary Figure 3 — PICRUSt analysis revealed that the metabolic pathways of butanoate metabolism and propanoate metabolism (highlighted in red boxes) might be relevant to EAP. n=10/group; EAP, experimental autoimmune prostatitis; PICRUSt, phylogenetic investigation of communities by reconstruction of unobserved states. [file Image_3.tif]

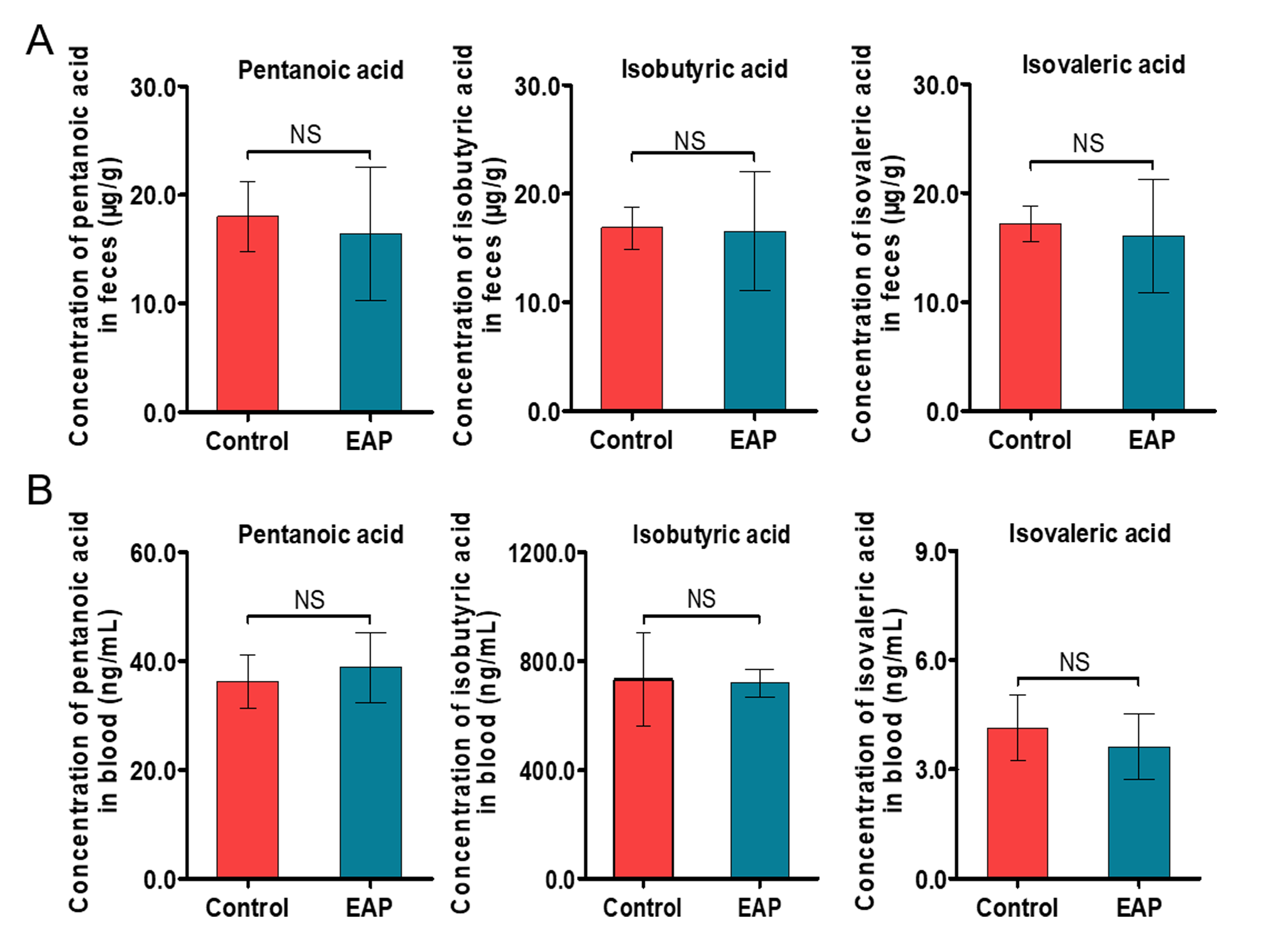

Supplement: Supplementary Figure 4 — Detection of the absolute concentrations of SCFAs, including pentanoic acid, isobutyric acid, and isovaleric acid, in control and EAP mice (A) The absolute concentrations of SCFAs, including pentanoic acid, isobutyric acid, and isovaleric acid, in feces from control and EAP mice. (B) The absolute concentrations of pentanoic acid, isobutyric acid, and isovaleric acid in serum from control and EAP mice. Data are the representative of three independent experiments. n=6-10/group; NS, not significant; EAP, experimental autoimmune prostatitis; SCFAs, short-chain fatty acids. [file Image_4.tif]

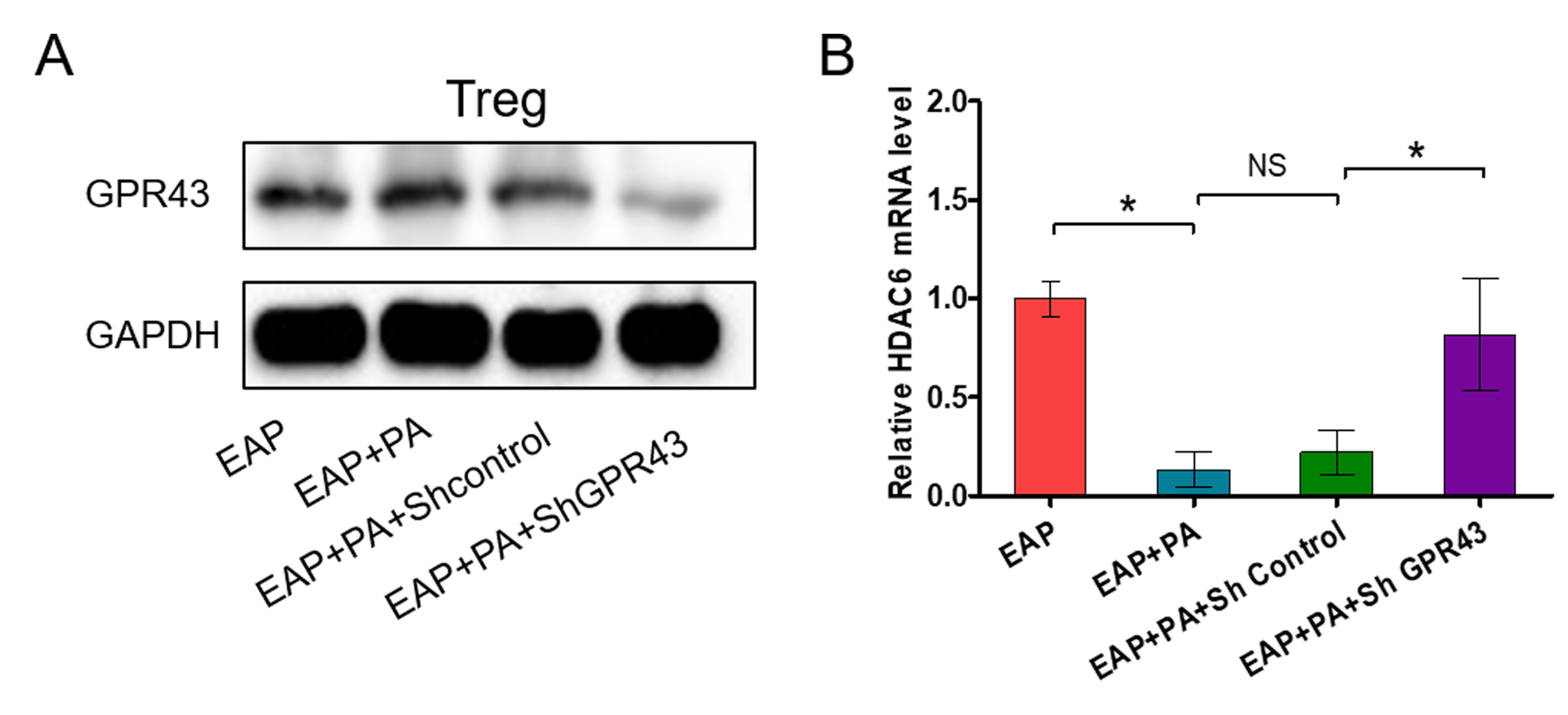

Supplement: Supplementary Figure 5 — Analysis of the GPR43 protein expression levels and the relative mRNA expressions of HDAC6 in different groups. (A) Immunoblot analysis of GPR43 expression levels in Treg cells that were isolated from mice of four groups: EAP group, EAP+PA group, EAP+PA+sh Control group, and EAP+PA+sh GPR43 group. (B) The relative mRNA expressions of HDAC6 in Treg cells that were isolated from mice of the four groups. n=3/group; *, P<0.05; NS, not significant; EAP, experimental autoimmune prostatitis; PA, propionic acid; GPR43, G-protein coupled receptors 43; HDAC6, histone deacetylase 6; Treg, regulatory T cell. [file Image_5.tif]
